# Supplementary figures and images for: Inhibition of SARS-CoV-2 polymerase by nucleotide analogs from a single-molecule perspective
Source: eLife. 2021 Oct 7;10:e70968. doi: 10.7554/eLife.70968 (PMC8497053; doi:10.7554/eLife.70968)

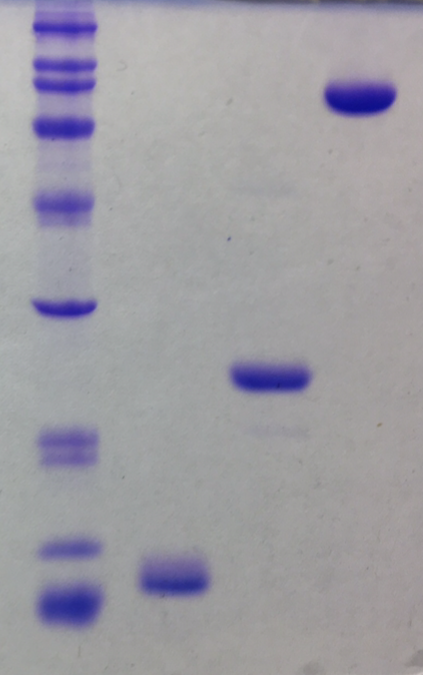

Supplement: Figure 1—figure supplement 1—source data 1. [file elife-70968-fig1-figsupp1-data1.zip › 200922 Gel of SARS CoV-2 nsp7 nsp8 nsp12 v2.png]

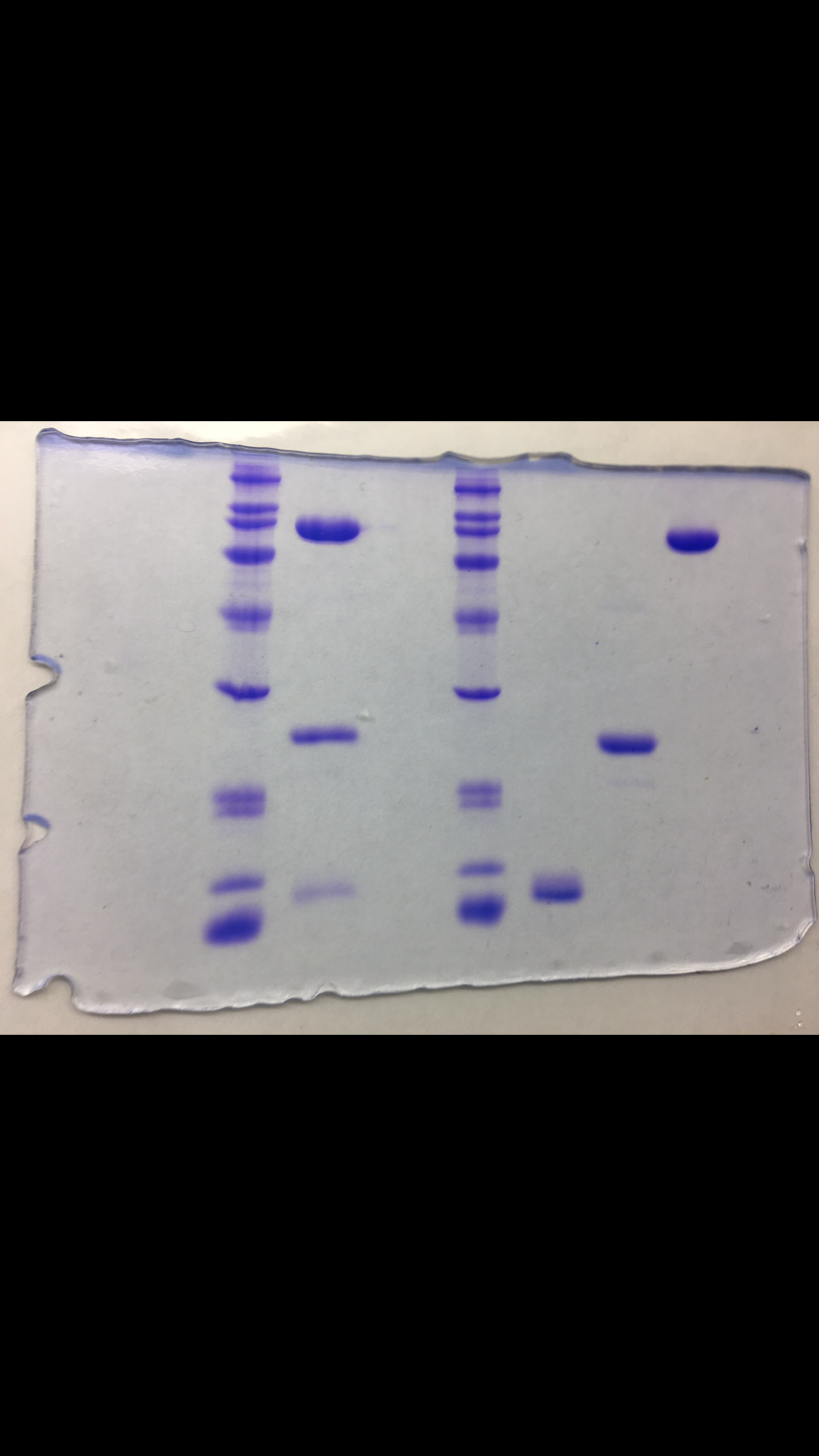

Supplement: Figure 1—figure supplement 1—source data 1. [file elife-70968-fig1-figsupp1-data1.zip › 200922 Gel of SARS CoV-2 nsp7 nsp8 nsp12 v1.png]

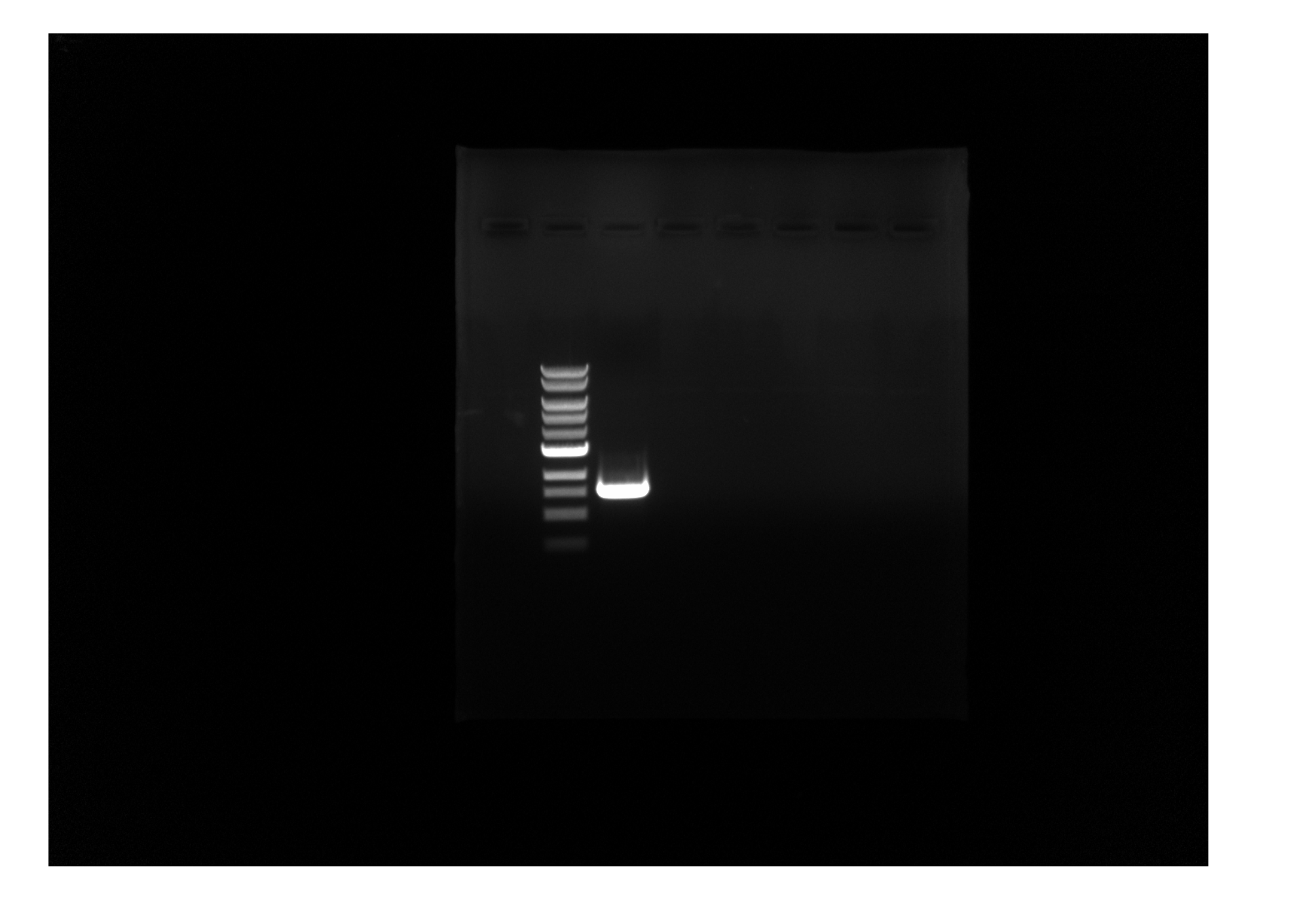

Supplement: Figure 6—figure supplement 4—source data 1. [file elife-70968-fig6-figsupp4-data1.zip › Figure_6_figure_supplement_4_source_data_1.tif]
